# Impact of Demineralization Time on Enamel Microhardness Reduction and Lesion Depth: An In Vitro Study

**DOI:** 10.7759/cureus.79441

**Published:** 2025-02-22

**Authors:** Murat Can Ersen, Zeynep Ceren Çelik, Melek Oztas, Merve Sahin, Dilek Tagtekin, Funda Yanikoglu

**Affiliations:** 1 Restorative Dentistry, Bursa Uludağ University, Bursa, TUR; 2 Faculty of Dentistry, Marmara University, Istanbul, TUR; 3 Restorative Dentistry, Kent University Faculty of Dentistry, Istanbul, TUR; 4 Restorative Dentistry, Marmara University Faculty of Dentistry, Istanbul, TUR

**Keywords:** demineralization, dental caries, enamel, lesion depth, microhardness

## Abstract

Introduction: The duration of acid exposure is a critical factor in determining an individual's risk of developing caries. The objective of this study was to investigate the correlation between demineralization time and two key parameters: microhardness and depth of an enamel caries lesion.

Methods: Sixty recently extracted human teeth were mounted in acrylic resin and randomly divided into five groups (n = 12). First, 400 grit and then 1,000 grit sandpaper were applied to the buccal surfaces for five minutes each. Other surfaces, except buccal surfaces, were coated with an acid-resistant varnish. The groups were stored in the same demineralization solution (pH 4.35-4.65), respectively, for a duration of 60, 72, 84, 96, and 108 hours. Before and after exposure to acidic solutions, the surface microhardness of samples (SMH) was evaluated using a Vickers pyramid diamond tip with a 100 gram load applied for 15 seconds. Three measurements were taken from each sample surface, and the average sample value was obtained by averaging the measurements. The depth of the lesions in three specimens from each group was evaluated from five different demineralized pits under a stereomicroscope. Data were analyzed by one-way ANOVA and post-hoc Tukey's honestly significant difference (HSD) tests.

Results: After acid exposure, SMH values significantly decreased in all groups (p = 0.001; p < 0.01) Statistically significant differences were shown between all groups in terms of Δhardness (p < 0.05). After 72 hours of demineralization, the microhardness values tend to decrease more as the exposure time increases (p = 0.001; p < 0.01). The mean lesion depths were varied from 4.01 ± 0.71 µm to 13.7 ± 1.17 µm.

Conclusion: Our findings show that there is a positive correlation between the duration of demineralization and lesion depth and a negative correlation between the duration of demineralization and microhardness. We assume that initial lesions may deepen quickly, especially after 72 hours.

## Introduction

The management of dental caries has experienced substantial changes in recent years. Modern strategies for caries management are based on the principles of early detection and prevention [1-3]. Furthermore, these strategies rely on the use of risk indicators and the assessment of risk factors, as highlighted in the literature [1,2].

The etiology of caries is multifactorial and results from the interaction of microbes, substrates, and host factors, including the anatomical features of the tooth surface that facilitate cariogenic microbial development. The proliferation and colonization of these microbes lead to plaque formation, characterized by an increase in acid-producing and acid-resistant bacteria, which disrupts the microbial balance of the biofilm. This imbalance can promote the fermentation of carbohydrates, producing acids in the oral cavity and resulting in a low pH. Ultimately, this process damages tooth structure through demineralization [4-8].

One of the primary protective mechanisms in the body is saturated saliva, which contains calcium and phosphate at a pH of 7, facilitating remineralization by depositing minerals in the porous regions where demineralization of enamel or dentin has started. This remineralization can occur if the oral cavity's pH remains sufficiently high for an adequate duration. However, if acidity persists, the demineralization process advances, leading to increased enamel porosity and ultimately resulting in the formation of carious lesions [9].

The formation of organic acids resulting from bacterial fermentation in the biofilm causes the pH value in the oral cavity to reduce below the critical pH values of 5.5 and 6.0 for enamel and dentin [10-12]. Under conditions where phosphoric acid and hydroxyl are below saturation levels, this allows the demineralization process of the tooth structure, allowing hydroxyapatite crystals to dissolve and form cavitation [7,10,11].

The concept of "critical pH" was introduced by Stephan [13] as a result of his research in this area. He asserted that the most effective way to relate pH changes to caries activity across different groups was to classify the values according to a "hypothetical critical decalcifying pH level." The critical pH of 5.5 was derived from the theoretical solubility of enamel in saliva and not in plaque fluid [14]. The critical pH denotes the threshold at which a solution reaches saturation concerning a specific mineral, such as tooth enamel. When the solution's pH exceeds this value, it becomes supersaturated, promoting mineral precipitation. Conversely, if the pH falls below the critical level, the solution remains unsaturated, leading to mineral dissolution until saturation is achieved [15].

Plaque bacteria metabolize fermentable carbohydrates, generating substantial amounts of acids, such as lactic acid. These acids become trapped between the tooth surface and the plaque biofilm, reducing the enamel pH below 5.6 and inducing enamel decalcification [16]. This process also leads to a substantial degradation in the mechanical properties of the affected tissues [17,18].

Enamel, the outermost layer of teeth, is the hardest tissue in the human body. The basic component of it is the enamel rod. The enamel rods consist of nano-fibrous hydroxyapatite (HA) crystals. It is composed of 96% hydroxyapatite (Ca10(PO4)6(OH)2) (HAp), 3% water, and 1% organic material. When exposed to acidic conditions (pH < 5.5), hydroxyapatite crystals dissolve, enamel rods and internal rods are destroyed, and enamel is demineralized. The critical pH at which this process occurs depends on various factors but is generally estimated to be around pH 5.5 [15].

Theoretical mathematical models have been proposed to describe the initial propagation and progression of caries; however, their predictive accuracy has yet to be validated through experimental evidence [19,20].

In vitro models are particularly appropriate for experiments designed to test a single process in isolation, as they allow for a more precise examination of the process under study, which might otherwise be affected by the presence of numerous variables. The composition of various demineralizing systems, including gels and solutions, has been designed to simulate the conditions of cariogenic biofilm during sugar metabolism. However, it is important to note that the calcium and phosphate concentrations, and in some cases, the pH values used in vitro are lower than those observed in the natural intraoral environment. This adjustment is made to accelerate demineralization compared to the in vivo process [21,22].

The microbiological component of dental caries is fundamental to the disease; however, its cariogenic potential is influenced by various factors. These include bacterial species, biofilm composition, saliva characteristics, and the quantity, type, and frequency of dietary carbohydrate consumption [23,24]. As the objective of this study was to characterize the process of acid demineralization of enamel, an acid-only caries model was employed.

The null hypothesis of this study was “there is no correlation between demineralization time and microhardness and lesion depth.”

## Materials and methods

This study was conducted with the approval of the Bursa Uludağ University Health Research Ethics Committee (Protocol No.: 2025-1/8). The preparation of extracted tooth samples was performed at Bursa Uludağ University Faculty of Dentistry, Bursa, Turkey, while the microhardness measurements and the acquisition of computerized surface images were carried out at Marmara University Faculty of Dentistry, Istanbul, Turkey.

The inclusion criteria are as follows: 1) Intact teeth: Only sound, non-carious permanent teeth were included to ensure uniformity in the enamel structure. 2) Extracted for orthodontic or periodontal reasons: Teeth extracted for reasons unrelated to caries or structural defects were included in the study. 3) No previous restorations or treatments: Teeth without previous fillings, sealants, or bleaching treatments were included in the study. 4) Similar age group: The age of donors was selected as 20-50 to minimize variations in enamel mineralization.

The exclusion criteria are as follows: 1) Teeth with existing enamel lesions: Teeth showing signs of hypocalcification, hypoplasia, erosion, fluorosis, or demineralization were excluded, as these conditions affect enamel hardness. 2) Caries or structural defects: The teeth with visible caries, cracks, or fractures were excluded. 3) Teeth with developmental anomalies: Teeth affected by conditions such as amelogenesis imperfecta or dentinogenesis imperfecta were excluded to avoid variability in enamel properties. 4) Teeth exposed to chemical agents: Teeth that had been exposed to fluoride treatments, acid etching, or remineralization agents before the study was excluded from the study.

Preparation of samples

In our study, a total of 60 caries-free human teeth, extracted for orthodontic and periodontal reasons, were utilized. The remaining soft tissue debris on the teeth was removed using a scalpel under running water. Subsequently, the crowns of the teeth were embedded in acrylic resin, positioned to reach 1-2 mm below the enamel-cement junction. Each acrylic block was prepared to contain a single tooth. The buccal surfaces of the teeth were then polished with sandpaper, first using 400 grit and then 1000 grit for a total of 10 minutes, five minutes each. To ensure that only the buccal surfaces were subjected to demineralization and that healthy enamel remained on the other surfaces, the teeth were coated with an acid-resistant varnish that was not affected by the demineralization solution. During periods when the study was not being conducted, the teeth were stored in closed plastic containers filled with water at room temperature (24 °C). The prepared samples were randomly divided into groups with 12 samples in each group.

Surface microhardness measurement before demineralization

**Figure 1:**
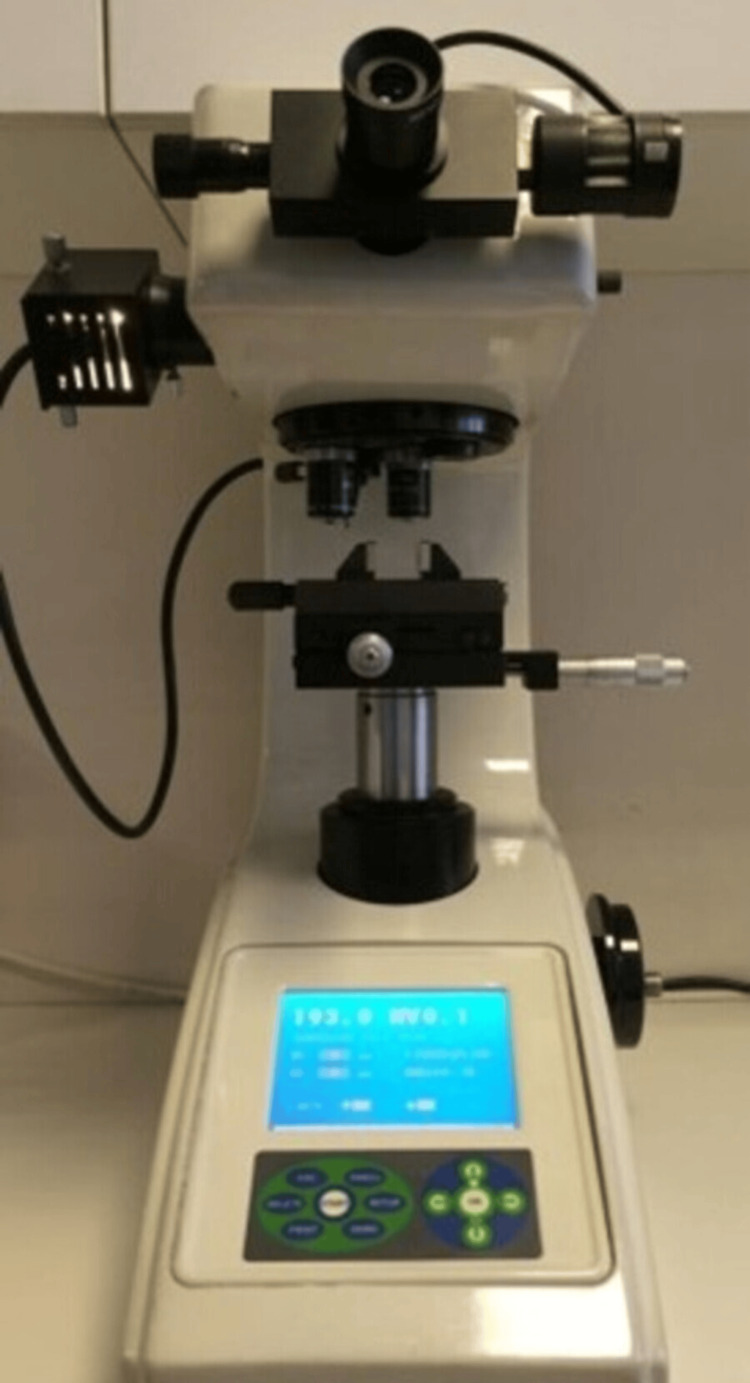
Application of Vickers diamond tip to samples in microhardness tester

**Figure 2:**
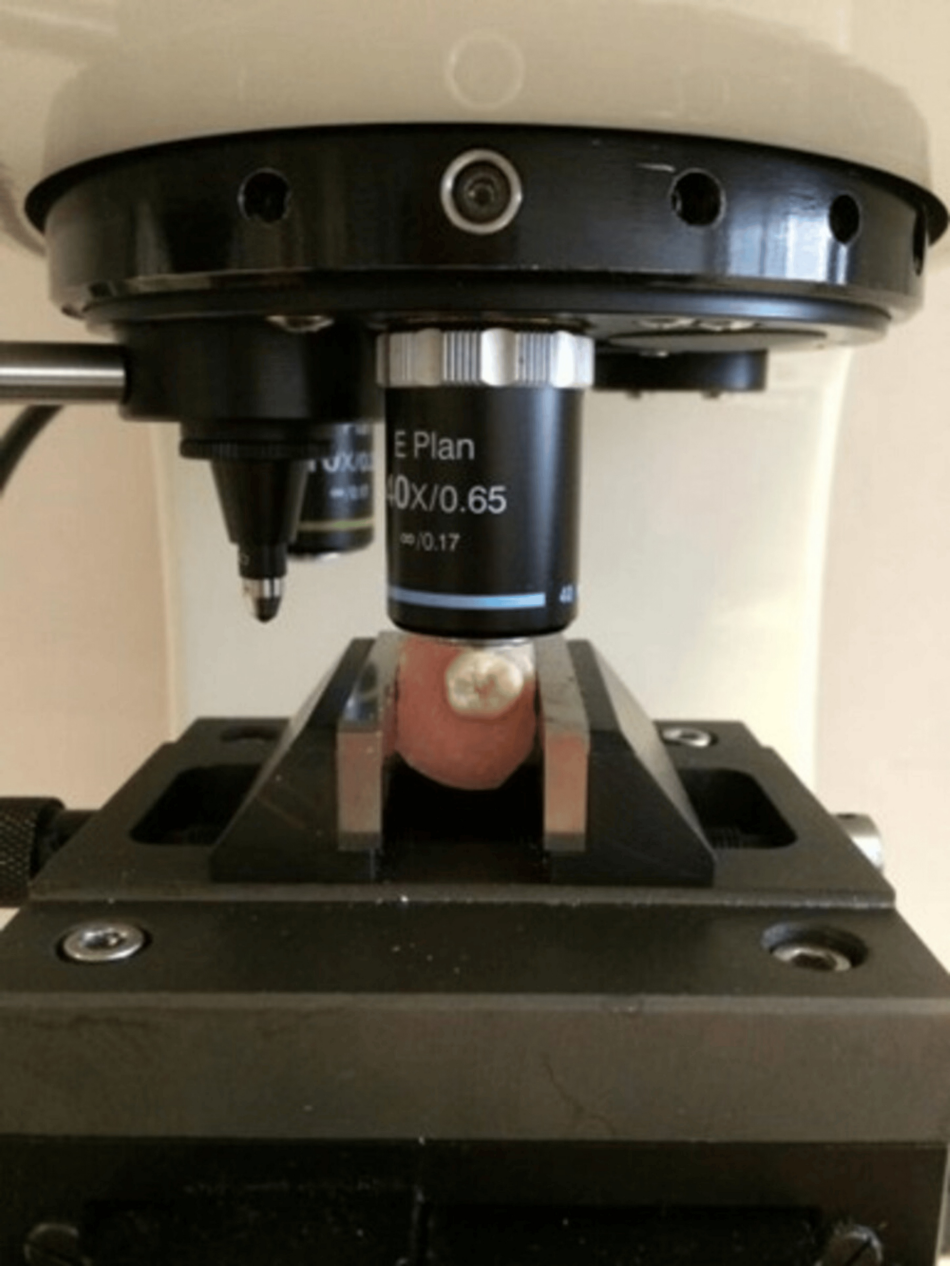
Examination of the marks created by the Vickers diamond tip in the microscope of the device

Samples were analyzed with a microhardness tester (Leitz Miniload Model LL, Wilson Mechanical Instrument Hardness Tester, Connecticut, USA) Vickers diamond tip was applied for 15 seconds with a 100 g load (Figure 1). Then, the mark obtained on the enamel surface was measured and evaluated with the microscope of the device (Figure 2). Microhardness values were obtained in Vickers Hardness (HV) units. Initial microhardness values of all samples were measured previously to demineralization solution exposure. Each sample surface was measured three times. Then, these three measurements were averaged, and the average sample value was obtained. In total, 60 x 3 = 180 measurements were made. The average value of each group was taken, and the group microhardness value was obtained.

Sample preparation

The teeth used in our study were disinfected in 0.1% thymol solution for 12 hours in a glass container in the refrigerator at +4 C for 12 hours before being kept in the demineralization solution. A 0.1% thymol solution is a solution that provides an antimicrobial effect without showing destructive effects on the histological results of artificial demineralization solution.

The demineralization solution consists of sodium acetate (0,1 mM CH_3_COONa), potassium chloride (150 mM KCl), calcium chloride (1,5 mM CaCl_2_), and potassium dihydrogen phosphate (0,9 mM KH_2_PO_4_).

Group 1 for 60 hours, Group 2 for 72 hours, Group 3 for 84 hours, Group 4 for 96 hours, Group 5 for 108 hours were kept in demineralization solution in a plastic container at room temperature (24 °C) for changing the solution every 24 hours.

Surface microhardness measurement after demineralization

After demineralization, measurements were taken again from the first measured areas of the teeth, and hardness values were recorded.

Sectioning demineralized teeth

In order to see the lesion depth of the demineralized teeth, the teeth were sectioned under water cooling in the cutting device (ISOMET 1000 Precision Saw, Buehler) in the R&D laboratory of Marmara University Faculty of Dentistry.

Examination and visualization of the sections taken in the stereomicroscope device

The sections were examined with a stereomicroscope (Leica M 27.5 Heeburg, Switzerland) in the R&D laboratory of Marmara University Faculty of Dentistry and computerized images were taken.

Statistical analysis

IBM SPSS Statistics for Windows, Version 22.0 (released 2013, IBM Corp., Armonk, NY) was used for statistical analyses in the evaluation of the findings obtained in the study. The suitability of the parameters for normal distribution was evaluated by the Shapiro-Wilk test, and it was found that the parameters were normally distributed. One-way ANOVA test was used for intergroup comparisons of the parameters and Tukey's honestly significant difference (HSD) test was used to determine the group causing the difference. Paired sample t-test was used in intragroup evaluations. Significance was evaluated at p < 0.05. level.

## Results

The initial mean microhardness values (VH) of the samples and the mean microhardness values (VH) after demineralization according to the groups are shown in Table 1. The decrease observed after demineralization compared to the initial microhardness level in all groups is statistically significant.

**Table 1:** Mean microhardness values at initial and after demineralization (VH) Groups formed according to the demineralization time: Group 1 was exposed to the demineralization solution for 60 hours, Group 2 for 72 hours, Group 3 for 84 hours, Group 4 for 96 hours, and Group 5 for 108 hours. **p < 0.01, statistically significant difference (paired sample t-test).

	Mean microhardness		P
	Initial	After demineralization	
	Mean ± SD (VH)	Mean ± SD (VH)	
Group 1 (60 h)	313.1 ± 0	261.64 ± 21.36	0.001**
Group 2 (72 h)	324.7 ± 0	270.20 ± 25.65	0.001**
Group 3 (84 h)	294.4 ± 0	236.36 ± 22.87	0.001**
Group 4 (96 h)	296.2 ± 0	218.80 ± 18.36	0.001**
Group 5 (108 h)	307.7 ± 0	226.16 ± 22.96	0.001**

The differences between the initial mean microhardness values of the samples in each group and the mean microhardness values measured after demineralization were expressed as Δhardness. Δhardness values according to groups are as follows: Group 1: 51.46, Group 2: 54.50, Group 3: 58.04, Group 4: 75.13, Group 5: 81.54 (Table 2). There is a statistically significant difference between the mean ∆hardness of the groups (p = 0.006; p < 0.01). As a result of the post-hoc Tukey's HSD test performed to determine which group the difference originated from, the mean ∆hardness of Group 5 was found to be statistically significantly higher than Group I (p = 0.018) and Group II (p = 0.042) (p < 0.05). There was no significant difference between the other groups in terms of ∆hardness averages (p > 0.05).

**Table 2:** ∆ microhardness values of the groups (VH) Groups formed according to the demineralization time: Group 1 was exposed to the demineralization solution for 60 hours, Group 2 for 72 hours, Group 3 for 84 hours, Group 4 for 96 hours, and Group 5 for 108 hours. A one-way ANOVA test was applied. **p < 0.01 indicates a statistically significant difference.

	∆ microhardness	
	Mean ± SD	P
Group 1 (60 h)	51.46 ± 21.36	0.006**
Group 2 (72 h)	54.50 ± 25.65	
Group 3 (84 h)	58.04 ± 22.87	
Group 4 (96 h)	75.13 ± 21.42	
Group 5 (108 h)	81.54 ± 22.96	

Mean lesion depths (µm) obtained by a stereomicroscope (Leica M 27.5 Heeburg, Switzerland) examination according to groups: Group 1: 4.01 ± 0.71, Group 2: 5.71 ± 0.73, Group 3: 6.98 ± 0.61, Group 4: 7.54 ± 1.33, and Group 5: 13.7 ± 1.16 (Table 3). A statistically significant difference exists among all groups except for Group III and Group IV.

**Table 3:** Mean lesion depths of the groups (µm) Groups formed according to the demineralization time: Group 1 was exposed to the demineralization solution for 60 hours, Group 2 for 72 hours, Group 3 for 84 hours, Group 4 for 96 hours, and Group 5 for 108 hours. A one-way ANOVA test was applied. **p < 0.05 indicates a statistically significant difference.

	Mean lesion depth	
	Mean ± SD	P
Group 1 (60 h)	4.01 ± 0.71 (a)	0.001 (a-b)
Group 2 (72 h)	5.71 ± 0.73 (b)	0.003 (b-c)
Group 3 (84 h)	6.98 ± 0.61 (c)	0.07 (c-d)
Group 4 (96 h)	7.54 ± 1.33 (d)	<0.001 (d-e)
Group 5 (108 h)	13.7 ± 1.16 (e)	<0.001 (a-d)

## Discussion

In our study, a demineralization solution was applied to the enamel surfaces to simulate artificial caries. The microhardness values of these surfaces were obtained before and after this procedure to determine the microhardness change during the demineralization process. One sample from each group was examined under a stereomicroscope, and lesion depths were measured. The null hypothesis was rejected: "There is no correlation between the demineralization time and microhardness and lesion depth."

Dental caries is a degenerative disease that affects the hard tissues of the tooth. It is caused by the acidic action of bacteria that are normally present within the oral cavity [25]. Since an acidic environment is necessary for the formation of dental caries, we evaluated the change in the enamel surfaces exposed to an acidic environment and the effect of the duration of exposure to an acidic environment on the amount of change in the enamel surface.

The specimens were subjected to demineralization in a solution prepared in a manner analogous to that applied in previous studies to obtain an artificial caries surface [26-28]. In accordance with the methodology proposed by Pulido et al., artificial carious lesions were developed by individually immersing acrylic-mounted enamel specimens in a continuously renewed demineralization solution, which was refreshed daily. In several studies, while CaCl_2_ is utilized in the demineralization solutions similar to that of our study, different compositions are also noted. Despite these variations in content, the pH value has been standardized at 4.4 [29,30].

 It was found that the teeth were kept in the demineralization solution for 72 hours, and demineralized enamel was formed at a depth of 25-50 microns [31]. Therefore, the hours in our study were organized according to these criteria.

Hardness is commonly defined as the "resistance of a material to deformation" [32]. Microhardness is closely related to the compositional properties of the materials under test and is influenced by reactions of the material surface [33]. Microhardness testing is a simple and reliable method for indirectly assessing changes in the mineral content of hard dental tissues and is utilized to evaluate the mechanical changes on the surface. Vickers surface microhardness technique has been used as an indirect method to assess mineral content in various laboratory models that simulate changes in the surface in vitro [34,35]. Microhardness measurements are performed using Vickers indenter, which is an indentation test that can provide a good determination of resistance to localized plastic deformation [36]. In this study, surface microhardness values were performed based on the Vickers microhardness test method.

The principal component of enamel is hydroxyapatite, which is susceptible to demineralization when exposed to acids. This process leads to the loss of essential minerals, including calcium and phosphate. The loss of minerals from the enamel results in a reduction in its strength, thereby increasing its susceptibility to damage [37]. Furthermore, extended exposure to acidic solutions results in elevated surface roughness and abraded depth values, accompanied by diminished microhardness [38-40].

The results of our study reveal a reduction in microhardness values for all groups subjected to demineralization over a period of 60 to 108 hours. Larnani et al. reported that even a mere one-hour exposure to an acidic solution resulted in a notable reduction in microhardness [41].

Bahrololoomi et al. evaluated the microhardness measurements of demineralized teeth in their study and reported a decrease of 67.57 ± 24.14 in the mean microhardness values of teeth kept in the demineralization solution for 72 hours. In our study, we recorded a decrease of 54.50 ± 25.65 in the mean microhardness value of the teeth kept in the demineralization solution for 72 hours. Despite observing comparable reductions, the numerical discrepancies may be attributed to variations in the composition of the demineralization solution [42].

In our study, the ∆hardness value was 81.54 in Group 5 after 108 hours of exposure to demineralization on the enamel surface, while in parallel with the results of our study, Elkassas et al. recorded a value of 88.17 after 108 hours in their study [43].

Cheng-fang Tang et al. examined the effect of grape seed on the remineralization of demineralized dentin. They first created artificial caries in the samples and examined the microhardness values. They reported that they obtained a significant difference between the control group and after demineralization with p < 0.05 [44]. In our study, there were statistically significant differences between initial and post-demineralization microhardness values in all groups.

Strnad and Buka reported that microhardness decreased with the duration of demineralization, as they examined enamel samples exposed to coke for five and 10 days and compared the changes in microhardness with the initial values. However, their study on the effect of acid erosion on enamel microhardness following remineralization revealed no significant differences. In our study, significant differences were observed between all groups. It was thought that the difference between both studies may be due to the difference in demineralization solutions [44].

Lata et al. first subjected the enamel samples to demineralization solution and measured the initial and post-demineralization microhardness values. The enamel samples were kept in a demineralization solution with a pH value below 5.5 for 72 hours. They reported a decrease of approximately 25 units in microhardness after demineralization. In our study, an average decrease of 54 units was found in the microhardness of the teeth kept in the demineralization solution with a pH value of 4.5 for 72 hours. As in our study, demineralization solutions that are more acidic may be more effective in decreasing the microhardness values [45].

When the ∆hardness values are analyzed, Group 5 was found to be statistically significantly higher than Group 1 (p = 0.018) and Group II (p = 0.042) These data show that there is a significant increase in ∆hardness values as the enamel exposure time to acidic environment increases.

After 72 hours of demineralization, microhardness values tend to decrease more as the exposure time increases (p = 0,001; p < 0.01). In agreement with our study, in the studies of Ferreira et al., the decreased tendency in microhardness values becomes more prominent as the exposure time increases [46]. Millet et al. also observed a slight decrease in the microhardness of demineralized samples in the first 24 hours, but a substantially greater decrease in microhardness values with increasing exposure time [47].

In our study, the samples were subjected to demineralization for a maximum of 108 hours, and a maximum lesion depth of 13 μm was observed. In the research conducted by Xue et al., both abraded and non-abraded surfaces were exposed to demineralization solutions for varying durations. Consistent with our findings on the depth of demineralized surface lesions, it was observed that, despite exposure times of up to 14 days, the demineralization depth on non-abrasive surfaces did not exceed 20 μm [48].

The main limitation of this study was the lack of standardization of the fluoride content in the enamel samples used. Although water fluoridation is not practiced in Turkey, natural variations in fluoride levels in drinking water in different regions, personal preventive measures, the patient’s age, and individual hygiene habits may directly influence the fluoride content of enamel and, consequently, its resistance to demineralization solutions. In future studies, the fluoride content of dental tissues could be measured using multiple proton microprobe analyses.

## Conclusions

The oral environment can become acidic due to multifactorial factors. Many variables such as dietary habits, systemic diseases, bacterial flora in the mouth, and frequency and quality of oral hygiene can lead to a long-term acidic oral environment. As a result, this threshold was determined to be 72 hours, indicating that after this point, demineralization accelerates significantly. This finding underscores the clinical importance of limiting prolonged exposure to acidic conditions to prevent severe enamel degradation.
